# Hematological investigations in a case of intravascular hemolysis due to *Clostridium perfringens* infection

**DOI:** 10.11613/BM.2024.021001

**Published:** 2024-04-15

**Authors:** Anamarija Beljan, Viktorija Blagec, Ana Bronic, Marina Pavić

**Affiliations:** Clinical Institute of Chemistry, Sestre milosrdnice University Hospital Center, Zagreb, Croatia

**Keywords:** gas gangrene, *Clostridium perfringens*, hip prosthesis, hemolysis, case report

## Abstract

A patient presented with fever, severe pain and edematous tight due to hip trauma and was scheduled for urgent fasciotomy. Following physical examination, laboratory analyses were requested, and results revealed anemia and severe infection. As the patient’s condition was serious, a new set of samples was sent to the laboratory four hours later. Following centrifugation, severely hemolyzed dark-colored serum and plasma samples were obtained and *in vitro* hemolysis was suspected. The collection of samples was repeated, but a new set of samples was also hemolyzed with a significant decrease in the hemoglobin value. At that point, *in vivo* hemolysis was suspected, and samples were processed according to standard laboratory procedures for hemolytic samples. Following confirmation of the gas gangrene diagnosis by clinicians, the cause of hemolysis was attributed to the cytotoxic activity of α-toxin produced by the anaerobic gram-positive bacterium *Clostridium perfringens.* An insight into the laboratory procedure that could help to narrow down the causes of hemolysis and single out *C. perfringens* as a cause of intravascular hemolysis was given.

## Graphical abstract

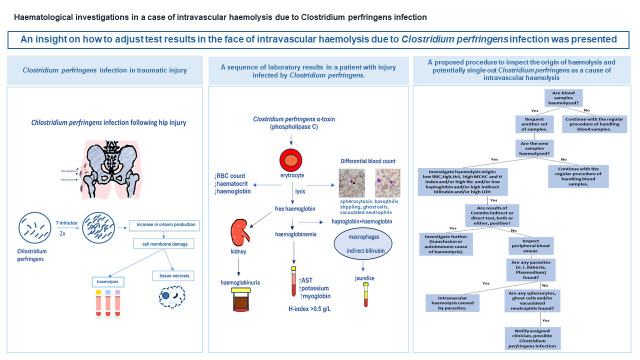

## Highlights

• *In vivo* hemolysis due to *C. perfringens* infection occurred in an extremely short period

• H index allowed monitoring severity of hemolysis and successful managing of samples

• Laboratory procedure to investigate origin of hemolysis and determine *C. perfringens* as a cause of intravascular hemolysis is presented

## Introduction

Gas gangrene is a rare but highly lethal complication of soft tissue infection with *Clostridium species*, most commonly *Clostridium perfringens (C. perfringens)*, accounting for 80-90% of post-traumatic infection cases (*1*). Traumatic injuries make way for bacteria to enter deep into tissue into an anaerobic environment suitable for rapid multiplication followed by toxin and gas production (*2*). *Clostridium perfringens* is a gram-positive bacteria found in soil as well as in human intestinal and genital microbiomes. Several types mainly cause enteric diseases, but type A is the most significant as it produces α-toxin and causes gas gangrene. *Clostridium perfringens* α-toxin (phospholipase C) hydrolyses membrane phospholipids into fatty acids causing damage to the membrane and ultimately, cell lysis resulting in hemolysis and necrosis. Massive intravascular hemolysis rarely occurs but carries a 70-100% chance of lethal outcome (*3*). Given the high mortality rate and median of 8 hours between admission to the hospital and fatal outcome, fast diagnosis is of great importance (*4*). Although blood or wound culture test remains a gold standard in differential diagnosis, it can take from 24 hours onwards to get a result, a period three times longer than the mortality median. Laboratory findings cannot provide a definitive diagnosis in such patients but can help in monitoring patient condition and the course of treatment and therapy. Here we describe a sequence of laboratory results in a patient with a hip injury infected by *C. perfringens* and give insight how to adjust test results in the face of intravascular hemolysis.

## Materials and methods

A patient presented with fever, severe pain and edematous tight due to hip trauma and was scheduled to urgent fasciotomy. Following physical examination, laboratory analyses were requested and during fasciotomy, wound culture specimens were collected for microbiological analysis. Further treatment included continuous monitoring in the emergency department and preparation of patient for another surgical procedure. No identifiable personal data were presented in this study. The patient deceased during hospitalization and therefore no written informed consent was obtained. The study was reviewed and approved by the Ethics Committee of Sestre Milosrdnice University Hospital Center (class: 003-06/23-03/022).

### Laboratory analyses

Blood samples were collected according to standard procedure and delivered to the laboratory immediately upon withdrawal. To determine complete blood count (CBC), blood samples were collected into lavander cap tube with anticoagulant dipotassium ethylene diamine tetra-acetic acid (K_2_EDTA) and analyzed at Sysmex XN1000 (Sysmex, Kobe, Japan). Blood smears were stained manually, using the May-Grunwald-Giemsa stain and were examined using Olympus BX43 (Olympus, Tokyo, Japan).

To obtain plasma for lactic acid determination and serum for all other biochemistry analyses, blood was collected into grey cap tube with sodium fluoride/potassium oxalate **(**NaF/KOx) and red cap tube with micronized silica particles cloth activator, respectively, and both tubes were centrifuged at 2100xg for 10 minutes. Myoglobin was analyzed on miniVIDAS (bioMérieux, Marcy I’Etolie, France), procalcitonin (PCT) on the Cobas e411 (Roche Diagnostics, Mannheim, Germany) whereas all other biochemistry parameters were determined on Architect c4000 (Abbott Laboratories, Abbot Park, IL, USA). Quantitative results of hemolysis and icterus indices for studied serum and plasma samples were measured on Architect c4000 by an automated spectrophotometric method which records the absorption at four different wavelength pairs, namely 500/524, 572/604, 628/660 and 524/804 nm, compared to 0.9% saline solution. Calculated concentrations of free hemoglobin and bilirubin are corrected for spectral overlap and results are reported in g/L of hemoglobin (H-index) and μmol/L for bilirubin (I-index).

Following blood collection into a blue cap tube with sodium citrate as anticoagulant and centrifugation at 2100xg for 15 minutes, routine coagulation assays were analyzed in plasma on Sysmex CS2500 analyzer (Sysmex, Kobe, Japan) and D-dimers on mini Vidas (bioMérieux, Marcy I’Etolie, France).

Urine was collected into standardized urine tube without preservative and the test strip was analyzed on the semi-automated Cobas u411 (Roche Diagnostics Ltd., Rotkreuz, Switzerland). Microscopic examination of urinary sediment was performed with Zeiss Axiostar Plus microscope (ZEISS, Göttingen, Germany) following sample centrifugation at 500xg for 8 minutes. All used tubes were designated by the manufacturer specification (Vacuette Greiner-Bio-One GmbH, Kremsmünster, Austria).

Arterial blood samples, collected into blood gas syringes (Westmed, New York, USA) were analyzed at ABL90 Flex analyzer (Radiometer Medical ApS, Brønshøj, Denmark). The most representative measurements of requested parameters on each day of follow-up are presented in Table 1.

**Table 1:** Results of complete blood count, biochemistry and coagulation assays measured the first six days after patient admitted to hospital

**Day of sampling**																		
	**1st day (admission)**			**2nd day**				**3rd day**			**4th day**		**5th day**			**5th day**		
**Sample number**																		
	**1**	**2**	**3**	**1**	**2**	**3**	**4**	**1**	**2**	**3**	**1**	**2**	**1**	**2**	**3**	**1**	**2**	**3**
**Assay (unit)**																		
RBC (x10^12^/L)	3.22	/	1.74	2.53	2.8	2.2	3.02	2.76	2.22	3.54	3.3	2.75	3.00	2.55	3.11	3.16	3.10	3.68
Hgb (g/L)	98	/	55^†^	81^†^	89^†^	70^†^	92^†^	86^†^	72^†^	106^†^	97^†^	83^†^	91^†^	78^†^	97^†^	98	96	113
Hct (L/L)	0.309	/	0.173	0.228	0.249	0.190	0.263	0.236	0.195	0.313	0.284	0.238	0.261	0.223	0.274	0.277	0.269	0.315
MCV (fL)	96.0	/	99.4	90.1	88.9	86.4	87.0	85.5	87.8	88.4	86.1	86.5	87.0	87.5	88.1	87.7	86.8	85.6
MCH (pg)	30.4	/	31.6^†^	32.0^†^	31.8^†^	31.8^†^	30.4^†^	31.2^†^	32.4^†^	29.9^†^	29.4^†^	30.2^†^	30.3^†^	30.6^†^	31.2^†^	31.0	31.0	30.7
MCHC (g/L)	317	/	318^†^	355^†^	357^†^	368^†^	349^†^	364^†^	369^†^	339^†^	342^†^	349^†^	349^†^	350^†^	354^†^	354	357	359
RDW (%)	14.9	/	15.7	16.5	16.0	15.9	14.7	14.7	14.9	14.3	14.6	14,9	15.2	15.5	15.1	15.1	15.2	14.7
PLT (x10^9^/L)	254	/	175	154	145	112	103	81	68	127	98	65	58	99	72	63	65	64
MPV (fL)	10.1	/	10.2	10.9	10.6	10.8	10.8	10.9	11.3	10.4	11.0	10.6	11.4	10.9	11.5	11.6	12.0	11.8
WBC (x10^9^/L)	30.6	/	34.8	29.8	29.7	25.6	27.9	26.7	18.0	22.1	23.4	17.3	15.7	14.4	11.7	12.6	12.4	13.2
TBIL (µmol/L)	NR	NR	NR	309	383	NR	NR	547	454	NR	426	NR	319	NR	287	282	258	NR
AST (U/L)	NR	NR	NR	156*	NR	NR	NR	126	95	NR	NR	NR	38	NR	NR	50	NR	NR
ALT (U/L)	NR	NR	NR	43*	NR	NR	NR	34	29	NR	NR	NR	30	NR	NR	44	NR	NR
CRP (mg/L)	260.6	NR	NR	247.8*	275.5*	NR	NR	317.6	285.0	NR	260.9	NR	176	NR	NR	189	212.3	NR
GGT (U/L)	NR	NR	NR	78*	NR	NR	NR	45	NR	NR	NR	NR	90	NR	NR	108	NR	NR
Na (mmol/L)	134	134	NR	135	137	NR	140	137	139	140	139	136	136	NR	NR	136	134	136
K (mmol/L)	5.6	/	5.9*	5.0*	5.2*	NR	5.8*	5.6	5.4	5.4	4.5	3.9	4.3	NR	NR	3.9	4.0	4.3
Cl (mmol/L)	101	NR	NR	105	109	NR	112	108	109	109	107	NR	103	NR	NR	103	103	NR
PCT (µg/L)	8.15	NR	NR	7.06	10.52	NR	NR	18.36	NR	NR	8.84	6.58	6.24	NR	5.07	4.69	NR	NR
Myo (µg/L)	9636	NR	NR	> 10,000*	NR	NR	NR	> 10,000	> 10,000	NR	NR	NR	2601	NR	NR	NR	NR	NR
H-index (g/L)^ǁ^	0.1	2.5	3.3	6.1	5.4	NR	1.8	0.5	0.0	0.1	0.0	0.0	0.0	NR	0.0	0.0	0.0	0.0
I-index (µmol/L)^ǁ^	122	143	162	222	288	NR	446	498	387	456	380	323	283	NR	249	246	224	239
Lact (mmol/L)	NR	/	NR	3.2*	1.9*	NR	1.1^§^	0.7^§^	0.9^§^	0.8^§^	0.8^§^	0.8^§^	0.8^§^	NR	NR	0.7	0.8	NR
H-index (g/L)^ǁ^	NR	2.6	NR	6.6	5.9	NR	1.8	0.3	0.0	0.0	0.0	0.0	0.0	NR	NR	0.0	0.0	NR
I-index (µmol/L)^ǁ^	NR	131	NR	197	255	NR	397	455	369	398	336	292	254	NR	NR	218	201	NR
PT (%)	75	/	61*	60*	61*	NR	56	72	67	75	90	NR	90	NR	NR	83	72	NR
PT INR	1.1	/	1.3*	1.3*	1.3*	NR	1.3	1.2	1.2	1.1	1.1	NR	1.1	NR	NR	1.1	1.2	NR
aPTT(s)	NR	/	23.0*	26.1*	26.6*	NR	30.0	30.2^§^	90.5^§^	28.8^§^	28.8^§^	NR	28.8^§^	NR	NR	31.9	68.1	NR
aPTT ratio	NR	/	0.9*	1.0*	1.1*	NR	1. 2	1.2^§^	3.6^§^	1.1^§^	1.1^§^	NR	1.1^§^	NR	NR	1.3	2.7	NR
Fibrinogen (g/L)	NR	/	>7.6*	/	/	NR	5.3	5.4	NR	5.0	6.3	NR	5.4	NR	NR	5.0	5.3	NR
Values marked with slash (/) were reported with interpretative comment: „Samples of whole blood, serum and plasma hemolyzed. Significant influence of hemolysis on assays marked with a „/“, an analysis couldn’t be performed.“ *Values reported as: „Samples hemolyzed and significant influence of hemolysis on assays. Results reported on clinician’s request.„ ^†^Values of hemoglobin, MCH and MCHC corrected. ^§^Values reported as: „Influence of icterus on assays. Results reported on clinician’s request.” ^ǁ^H-index was reported as g/L of hemoglobin and I-index as µmol/L of bilirubin. For all biochemistry analysis they were measured on Architect c4000 (Abbott Laboratories, Abbot Park, IL, USA) analyzer. Cutoff values of studied parameters for H-index (g/L) were as follows: for K, AST 0.5; ALT 1.5; GGT 2.5 ; CRP 5.0 ; albumin 7.5; PCT 9.0; Na, Cl, TBIL and creatinine 10.0; Myo 4.7 and Lact 1.9. Cutoff values for I-index (µmol/L) were: 1026 for electrolytes, AST, ALT, GGT and albumin; 1129 for CRP; 428 for PCT; 540 for Myo; 684 for creatinine and 217 for lactate. Interference of hemolysis or icterus for CBC and coagulation assays was detected due to occurrence of “ turbidity/Hgb“ flag on Sysmex XN1000 (Sysmex, Kobe, Japan) and „H“- flag by Sysmex CS2500 (Sysmex, Kobe, Japan) analyzer, respectively. According to the laboratory’s policy in both cases, samples are rejected and results could only be issued at the clinician’s request. NR - not requested. CBC - complete blood count. RBC – red blood cells. Hgb – hemoglobin. Hct – hematocrite. MCV – mean cellular volume. MCH – mean cellular hemoglobin. MCHC - mean cellular hemoglobin concentration. RDW – red blood cell distribution width. PLT – platelet. MPV – mean platelet volume. WBC – white blood cell count. TBIL – total bilirubin. AST – aspartate aminotransferase. ALT – alanine aminotransferase. CRP – C reactive protein. GGT – gamma glutamyl transferase. Na – sodium. K – potassium. Cl – chloride. Lact – lactate. PCT – procalcitonin. Myo – myoglobin. PT - prothrombin time. aPTT - activated partial thromboplastin time.																		

### Investigations and further interventions

Blood tests performed on the patient’s admission revealed anemia and leukocytosis that, in addition to a high concentration of C-reactive protein (CRP) and PCT, indicated severe infection (Table 1). Severe damage of muscle tissue was evident from a high concentration of myoglobin (Table 1) whereas high concentrations of creatinine (196 µmoL/L) with subsequently low estimated glomerular filtration rate (eGFR) of 21 mL/min/1.73m^2^ pointed to patient`s renal dysfunction.

Due to the patient`s serious condition, a new set of samples was sent to the laboratory four hours later. Upon centrifugation, severely hemolyzed, dark-colored serum and plasma samples were obtained. Nevertheless, all requested analysis were performed but as significant interference of hemolysis was assigned on potassium, lactate, coagulation assays and CBC, according to laboratory policy obtained results were not reported. Instead a character „/“ was entered in the column and an appropriate interpretative comment was added on the report: ”*Samples of whole blood, serum and plasma are hemolyzed. Significant influence of hemolysis on assays marked with a „/“, an analysis couldn’t be performed.“* As *in vitro* hemolysis was suspected, it was also suggested to the nurse to repeat the collection of the samples. As a new set of samples was hemolysed as well, all requested analysis were performed and results revealed a significant drop in hemoglobin, to half of the initial value (from 98 g/L to 55 g/L within a 4-hour interval), high concentration of potassium (5.9 mmol/L) as well as metabolic acidosis (pH 7.290, actual bicarbonate 20 mmol/L). Thus, the clinician was consulted regarding suspicion of intravascular hemolysis, and in agreement, it was decided to report the obtained values. However, before the release of the report, correction of the hemoglobin values, mean cellular hemoglobin (MCH) and mean cellular hemoglobin concentration (MCHC) results were mandatory due to hemolysis. Correction of values was performed according to standard procedure (*5*). The sample for CBC was centrifuged, and the value of hemoglobin obtained in the plasma was subtracted from the hemoglobin in the original run. Corrected value of the hemoglobin was used to calculate MCH and MCHC that were reported. A complete report was released with an interpretative comment (Table 1).

**Figure 1:**
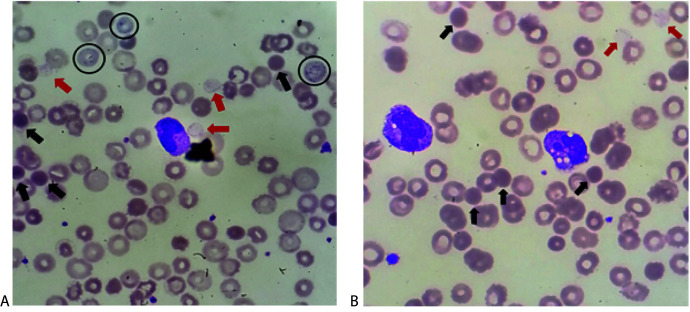
Microscopic images of the peripheral blood smear of the patient. Figure 1A. Microscopic images showing ghost cells (red arrows), spherocytes (black arrows), basophilic stippling of erytrocytes (circled black). Figure 1B. Microscopic image showing spherocytes (black arrows), ghost cells (red arrows) and two vacuolated neutrophils.

On the next day, hemolyzed samples were obtained as well, and laboratory results revealed a high value of total bilirubin, aspartate aminotransferase (AST) and gamma glutamyl transferase (GGT) along with a low albumin concentration of 17 g/L that indicated liver dysfunction. High concentration of fibrinogen, PCT and CRP pointed to severe infection (sepsis) and along with prolonged prothrombin time (PT), high D-dimer concentration (4.3 mg/L FEU) and low platelet count course toward disseminate intravascular coagulation. Results are presented in Table 1. May-Grünwald-Giemsa-stained peripheral blood smears showed spherocytes, hemoglobin-deprived erythrocytes varying in size, basophilic stippling of erythrocytes, nucleated red blood cells (NRBC) and ghost cells without a sign of schistocytes. The left shift was obvious with the dominant presence of band neutrophils, whereas most of neutrophils had vacuolated cytoplasm (Figure 1). No intravascular hemolysis-causing parasites, such as Plasmodium or Babesia, were found.

In addition to serum and plasma analyses, urine analysis was requested. However, a dark-colored brown urine sample was delivered to the laboratory. The urine dipstick was positive for bilirubin, protein, and hematuria without evidence of erythrocytes in urine sediment, which pointed to hemoglobinuria without hematuria (results not shown).

To investigate the probable origin of hemolysis, the indirect and direct antiglobulin tests were also performed and the results came out negative. In the meantime, wound cultures sampled on admission during the fasciotomy were positive for *C. perfringens*. Thus, the diagnosis of gas gangrene suspected by clinicians was confirmed and explained as the cause of intravascular hemolysis.

On a third day, all obtained samples were slightly hemolyzed and urine was still dark-colored, but samples became icteric and interference on the following parameters was observed: lactate, activated partial thromboplastin time (aPTT), hemoglobin, MCH and MCHC (Table 1). However, correction of the CBC results was performed due to icterus, by saline replacement procedure that involves centrifugation of the sample, removing the plasma, and replacing it with an equal amount of saline. The sample was re-mixed and run on the analyzer whereas according to the obtained value of hemoglobin, values of MCH and MCHC were corrected (*5*). All other CBC parameters were reported from the original run. Results were reported with an interpretative comment (Table 1).

However, a drop of standard inflammatory markers, leukocytes, CRP, PCT and fibrinogen was monitored until the fifth day of the patient’s hospitalization, whereas an increase in CRP was recorded on 6th day of hospitalization when also IL-6 value of 714 pg/L (normal value < 7 pg/L) was recorded. In addition, on the sixth day of hospitalization, the obtained samples were clear with no signs of hemolysis or icteria. H index determined in analyzed serum and plasma samples was in accordance with obtained results and showed the expected course (Table 1).

## What happened?

*Chlostridium perfringens* is an anaerobic gram-positive bacterium that can proliferate and increase toxin production in a very short time due to a doubling time of only 7 min. Therefore, it is not unusual that samples become hemolytic in a short period. Very low values of red blood cells (RBC) count and hematocrit paired up with measured hemoglobin values led to the subsequent mark-up of MCHC, all of it being a consequence of α-toxin’s hydrolytic activity and destruction of the RBC membrane. Specific findings such as spherocytosis, basophilic stippling, ghost cells, vacuolated neutrophils and left shift were reported on a peripheral blood smear result. Damage to the cell membrane causes RBC to swell and adopt a spherical shape and ultimately burst due to their higher osmotic fragility. Both spherocytes and ghost cells *i.e.*, burst RBC completely deprived of their intracellular content could be visible on a peripheral blood smear. Along with hemoglobin, AST, potassium and chloride ions released from damaged RBC resulting in a high concentration in blood serum samples.

Because of RBC destruction, hemoglobin but no RBC was found in urine. However, upon receiving the first hemolyzed set of samples, *in vitro* hemolysis was suspected. To eliminate the possibility of hemolysis being an error during venipuncture, a new set of samples was requested, but the obtained serum and plasma were severely hemolysed as well. Paired with dark, red-colored urine, the possibility of a pathological cause seemed more likely to be the origin of hemolysis. The direct and indirect Coombs tests were negative, and the results eliminated autoimmune hemolytic anemia or hemolytic transfusion reactions as causes of intravascular hemolysis.

However, wound culture results revealed *in vivo* origin of hemolysis. Besides α-toxin, *C. perfringens* produces θ-toxin (perfringolysin) which indirectly contributes to the development of septic shock by stimulating the release of IL-6 (*2*). In a state of severe sepsis, liver and renal functions are impaired which results in icteric samples and the patient undergoing hemodialysis.

In the present case, the infection was rapid and progressive, but the necrotizing course was controlled successfully by extensive monitoring, early surgery and appropriate antibiotic therapy. Thus, from the sixth day until the end of patient hospitalization interfering components also were not present in laboratory samples.

## Discussion

Correct differentiation between *in vivo* and *in vitro* hemolysis is of great clinical importance as *in vivo* hemolysis could be a sign of many different underlying pathological conditions, some of which could be life-threatening if left untreated. In our case, *in vivo* hemolysis was the consequence of gas gangrene - a very serious condition with potential complications such as permanent tissue damage, infection, liver damage, kidney failure, shock, coma and even death, all within only a couple of hours after symptoms occur. Obtained values of myoglobin indicated severe damage of muscle tissue that coincides with *C. perfringens*-induced tissue necrosis whereas its consequence, severe hemolysis, interfered and affected analytical determination of common laboratory parameters (*4*, *6*-*13*). Interestingly, hemolyzed samples appeared due to second sampling, four hours after obtaining of first set of clear samples. Numerous cases mention a second measurement of hemoglobin or hematocrit within 24 hours of admission: in > 80% of cases obtained values decreased to those less than half of the initial ones, as it was in our case (*4*). Besides hemolyzed blood samples, dark-colored urine samples with hemoglobinuria and without hematuria was also reported just like it was in our patient (*14*).

However, when hemolytic samples are obtained, it is important to investigate the degree of hemolysis and report results as soon as possible, to avoid delays in patient follow-up and safety (*15*). The degree of hemolysis in our study was monitored for all serum and plasma samples by the H index that showed an expected course of values which followed the patient`s clinical condition. Moreover, measuring the H index allowed us precise determination of cell-free hemoglobin, the severity of hemolysis and the correct mark-up of requested parameters affected by interference as shown in Table 1. As there was no possibility of obtaining non-hemolyzed samples due to hemolysis *in vivo*, the decision to report affected results was made after consultation with the clinician. However, these results were released with an interpretative comment by means to be interpreted within a clinical context. Following clinical interventions, hemolysis was resolved on the third day, but icteric samples were obtained due to an increase in bilirubin concentration. As the cut-off on CBC (hemoglobin, MCH, MCHC), lactate and aPTT for icteria was exceeded, results were reported with an interpretative comment, too, by means to be interpreted within the clinical context.

There is no gold standard test to confirm *in vivo* hemolysis, and in addition to a patient’s history, laboratories most often rely on laboratory assays such as increased reticulocyte count, haptoglobin, indirect bilirubin or lactate dehydrogenase (LDH). On the other hand, these parameters are not sensitive enough and could be present in conditions other than hemolysis that may confound the clinical picture (*7*). For example, reduced concentrations of haptoglobin are observed in conditions other than hemolysis, such as liver impairment or malnutrition. Furthermore, haptoglobin increases in inflammatory diseases possibly masking an underlying hemolytic condition. However, if available, the test should be performed to acquire as much as possible evidence of intravascular hemolysis. The limitation of our study lies in the unavailability of a haptoglobin assay in our laboratory. Moreover, indirect bilirubin was sent to determination in the collaborating laboratory but was not reported due to exceeding the H index threshold. As gas gangrene diagnosis was at the time already established, we did not investigate further its value. The definitive diagnosis of intravascular hemolysis in this study came after confirmation of wound culture test of *C. perfringes* infection and made easier managing of further samples for laboratory personnel as well as interpretation of obtained results for clinicians.

The main takeaways of our investigation lie in RBC parameters and peripheral blood smear findings which are very consistent with the ones found in the reviewed literature, research, and case studies. As in our case severe hemolytic anemia, the presence of spherocytes, ghost cells, and vacuolated neutrophils with evident left shift and without evidence of schistocytes or parasites in peripheral blood smears are a repeating motif in most of the reviewed cases and should unequivocally raise suspicion of *C. perfringens* infection (*3*, *4*, *8*, *16*-*21*). The possibility of finding gram-positive bacilli in the direct gram stain of a patient’s peripheral blood or even bacilli englobed by neutrophil in a regular Giemsa-stained peripheral blood smear was reported but only vacuolated neutrophils in our study were observed (*8*, *16*).

Considering gas gangrene is an extremely rare condition and studies describing laboratory findings and proposed indicators or procedures for handling such a difficult case are scarce, an insight into the laboratory procedure that could help to narrow down the causes of hemolysis and aid the diagnostic process until treatment initiation is the main advantage of this study.

In conclusion, *C. perfringens* infection should be kept in mind when considering trauma patients with fever and severe hemolytic anemia, hemolytic serum/plasma samples and dark urine samples.

## What should/can you do in your laboratory?

When hemolyzed blood samples are administered to the laboratory, we propose taking the following steps to differentiate between hemolysis origins and further investigate the possibility of *C. perfringens* infection. The first call should be a request for another set of samples to be delivered to the laboratory immediately upon collection. If another set of hemolyzed samples is acquired, and moreover, dark urine obtained blood tests should be run and the origin of hemolysis should be investigated. The degree of hemolysis should be monitored by the H index and possibilities as well as limitations of the analysis in such samples should be discussed with the clinician. If available, changes in haptoglobin, reticulocyte, LDH and indirect bilirubin should be considered, and Coombs tests could be helpful too. The following changes in test results should be looked at hemolytic anemia, and when examining peripheral blood smear, spherocytes, ghost cells and vacuolated neutrophils should be observed.

**Figure 2:**
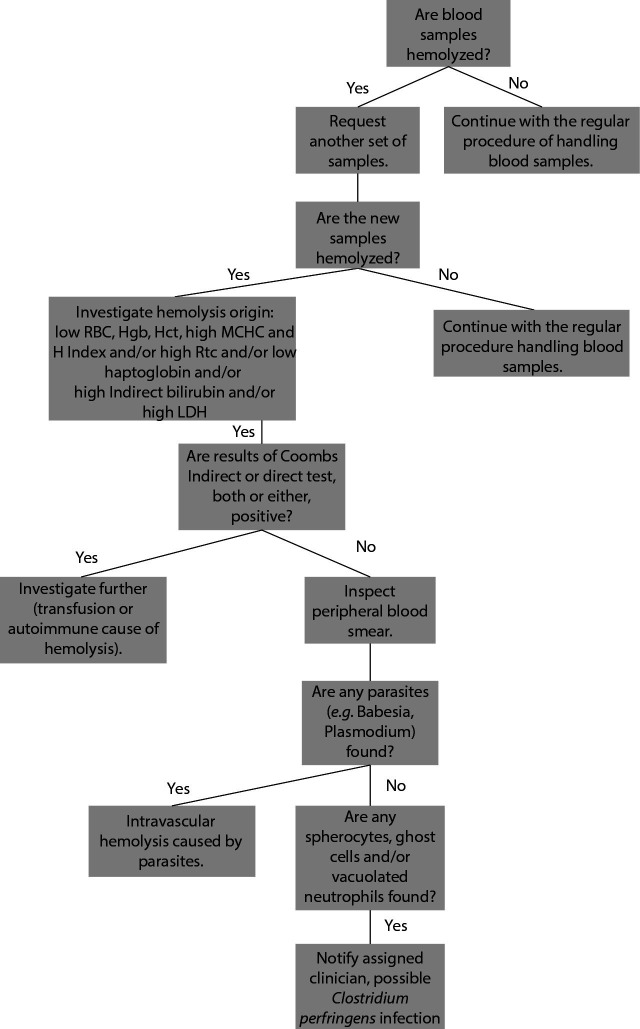
A proposed laboratory procedure to inspect the origin of hemolysis and potentially single out *Clostridium perfringens* as a cause of intravascular hemolysis. RBC - red blood cell. Hgb – hemoglobin. Hct – hematocrite. MCHC - mean cellular hemoglobin concentration. H index - hemolysis index. Rtc – reticulocyte. LDH - lactate dehydrogenase.

A proposed laboratory procedure to inspect the origin of hemolysis and potentially single out *C. perfringens* as a cause of intravascular hemolysis is presented in Figure 2.

## Data Availability

All data generated and analyzed in the presented study are included in this article.
